# Occurrence of PCDD/Fs and PCBs in Edible Land Snails (*Helix pomatia*) from Poland: Tissue Distribution and Implications for Food Safety

**DOI:** 10.3390/toxics14070599

**Published:** 2026-07-09

**Authors:** Monika Ziomek, Julia Kowalczyk, Marek Pajurek, Szczepan Mikołajczyk, Wojciech Pietroń, Krzysztof Szkucik, Michał Gondek

**Affiliations:** 1 Department of Food Hygiene of Animal Origin, Faculty of Veterinary Medicine, University of Life Sciences in Lublin, Akademicka 12, 20-033 Lublin, Poland; monika.ziomek@up.edu.pl (M.Z.); julia.kowalczyk@up.edu.pl (J.K.); krzysztof.szkucik@up.edu.pl (K.S.); 2 Department of Chemical Research of Food and Feed, National Veterinary Research Institute, 57 Partyzantow Avenue, 24-100 Pulawy, Poland; marek.pajurek@piwet.pulawy.pl (M.P.); szczepan.mikolajczyk@piwet.pulawy.pl (S.M.); wojciech.pietron@piwet.pulawy.pl (W.P.)

**Keywords:** PCDD/Fs, PCBs, terrestrial gastropods, *Helix pomatia*, food safety, bioaccumulation

## Abstract

Terrestrial snails are recognised as bioindicators of environmental contamination and may represent a source of dietary exposure to persistent organic pollutants (POPs). However, data on polychlorinated dibenzo-*p*-dioxins, dibenzofurans (PCDD/Fs), and polychlorinated biphenyls (PCBs) in edible land snails remain limited. This study investigated PCDD/Fs, dioxin-like PCBs (dl-PCBs), and non-dioxin-like PCBs (ndl-PCBs) in the foot muscle and hepatopancreas of free-living *Helix pomatia* (*H. pomatia*) collected from two regions of Poland. A total of 48 snails (24 per region) were analysed as three pooled replicates per tissue type in each region. Thirty-five congeners were determined using high-resolution gas chromatography coupled with high-resolution mass spectrometry (HRGC-HRMS). Overall, only trace concentrations of selected PCDFs and dl-PCBs were detected, exclusively in hepatopancreas samples. Among PCDFs, only 2,3,7,8-TCDF and 1,2,3,7,8-PeCDF were quantified, whereas PCB 77 was the only quantified dl-PCB congener. All analysed PCDD congeners and ndl-PCBs were below the limit of quantification. TEQ values calculated using 2005 WHO-TEFs remained very low in all analysed samples, including under the upper-bound approach. The preferential detection of contaminants in the hepatopancreas was consistent with the higher lipid content of this tissue. The results indicate very low contamination of *H. pomatia* with dioxins and PCBs and low toxicological relevance under the investigated conditions.

## 1. Introduction

Polychlorinated dibenzo-*p*-dioxins (PCDDs), polychlorinated dibenzofurans (PCDFs), and polychlorinated biphenyls (PCBs) are persistent organic pollutants (POPs) of considerable environmental and toxicological concern. Due to their persistence and resistance to degradation, these compounds are widely distributed in environmental compartments and may accumulate in biota [1,2,3,4,5]. The toxic effects of PCDD/Fs and dioxin-like PCBs (dl-PCBs) are primarily mediated through activation of the aryl hydrocarbon receptor (AhR). Epidemiological studies, particularly those involving exposure to 2,3,7,8-tetrachlorodibenzo-*p*-dioxin (TCDD), have linked these compounds to carcinogenic, immunotoxic, endocrine-disrupting, and developmental effects [6,7,8,9].

Land snails are widely recognized as useful bioindicators of environmental contamination due to their close association with soil and their feeding behaviour, which includes both soil-derived material and vegetation. Their capacity to accumulate contaminants has been extensively documented, particularly for trace metals, in numerous terrestrial gastropod species such as *Helix pomatia*, *Helix aspersa*, *Cepaea nemoralis*, *Eobania vermiculata*, *Papillifera papillaris*, and *Cantareus apertus* [10,11,12,13,14,15,16]. In addition to inorganic contaminants, selected species have also been shown to accumulate organic pollutants, including polycyclic aromatic hydrocarbons (PAHs) and pesticides, as demonstrated for *Helix aspersa* [17,18].

The consumption of terrestrial snails in many regions of the world raises concerns regarding their potential contribution to human exposure to environmental contaminants. In some cases, concentrations of lead and cadmium reported in edible snail tissues were comparable to or higher than those observed in animal-derived food products regulated under European Union legislation, including marine molluscs, which are among the food categories with the highest permissible contaminant levels [19,20,21].

Most available data on PCDD/Fs and dioxin-like compounds in gastropods concern marine and freshwater species, which have been extensively studied to assess contamination levels, potential risks for consumers, and their suitability as bioindicators of environmental pollution [22,23,24,25,26,27,28,29,30,31,32,33,34]. Studies on these aquatic gastropods have shown that contaminant burdens reflect local anthropogenic pressures. For example, elevated concentrations of organochlorine contaminants were observed in molluscs inhabiting areas affected by stormwater discharges and urban runoff [22], while higher PCB concentrations in marine gastropods were associated with the vicinity of former capacitor and paint manufacturing facilities [25]. In freshwater snails, PCB and PBDE concentrations decreased with increasing distance from e-waste recycling sites and were positively correlated with contaminant levels in surrounding soils [27]. In contrast, information on terrestrial gastropods remains extremely limited, particularly with respect to PCDD/Fs, for which data are virtually absent at the global level. This scarcity of data may reflect the historical focus of PCDD/F research on aquatic ecosystems and aquatic food webs, where these highly hydrophobic contaminants are known to accumulate in sediments and biota [35]. In addition, terrestrial gastropods have rarely been included in monitoring programmes targeting dioxin-like compounds. The possibility of generally low background concentrations in many terrestrial environments may have further limited research in this field. Moreover, determination of PCDD/Fs at trace levels is analytically challenging and often requires highly sensitive and selective techniques. In this context, HRGC-HRMS is widely regarded as the reference (gold standard) method for the congener-specific determination of PCDD/Fs at ultra-trace concentrations [36]. Nevertheless, recent studies suggest that land snails may accumulate selected PCB congeners, including dioxin-like compounds. For example, *Cornu aspersum* has been identified as a useful sentinel organism for biomonitoring of PCBs, reflecting their accumulation from soil and atmospheric deposition under varying degrees of anthropogenic pressure [37].

The distribution of contaminants among different snail tissues is also of particular importance. The foot muscle represents the principal edible part of the land snail and is therefore the most relevant tissue for assessing potential dietary exposure. In contrast, the hepatopancreas is a metabolically active organ involved in contaminant accumulation and detoxification processes [38]. This distinction is reflected in European Union legislation, which requires the removal of the hepatopancreas from edible snails if it may pose a hazard to human health [39]. However, terrestrial edible snails are not included as a separate food category under Commission Regulation (EU) 2023/915, and no maximum levels have been established for PCDD/Fs and PCBs in these organisms [40]. Given the absence of specific regulatory limits, the scarcity of available data, and the continued consumption of terrestrial snails in several European countries, including Poland, information on the occurrence of these contaminants remains important from both environmental biomonitoring and food safety perspectives, particularly in light of the growing interest in alternative and sustainable protein sources.

Against this background, the present study aimed to determine the concentrations of PCDD/Fs, dl-PCBs, and selected ndl-PCBs in edible (foot muscle) and non-edible (hepatopancreas) tissues of free-living *Helix pomatia* (*H. pomatia*) collected from two Polish voivodeships representing contrasting environmental and anthropogenic settings. Analyses were performed using high-resolution gas chromatography coupled with high-resolution mass spectrometry (HRGC-HRMS), and WHO-TEQ values were calculated to facilitate the toxicological interpretation of the results.

## 2. Materials and Methods

### 2.1. Sample Collection and Processing

**Figure 1:**
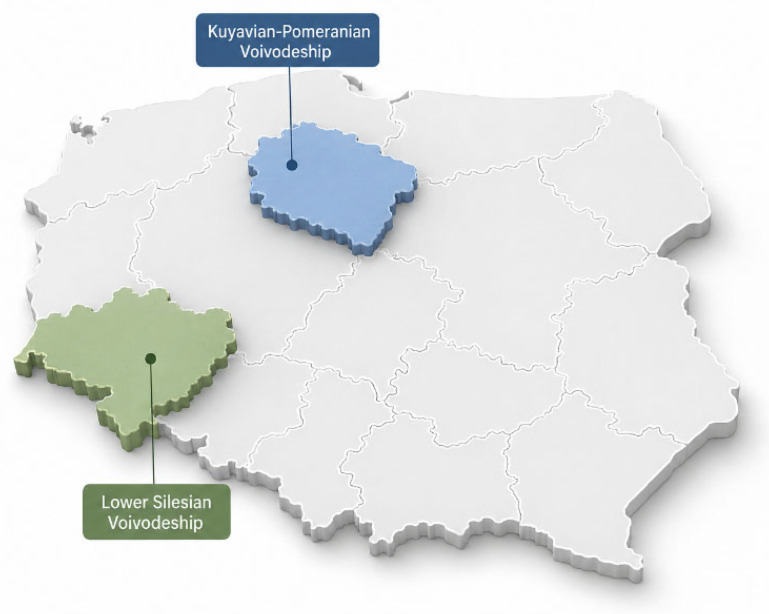
Voivodeships of origin of *Helix pomatia* specimens included in the study.

**Figure 2:**
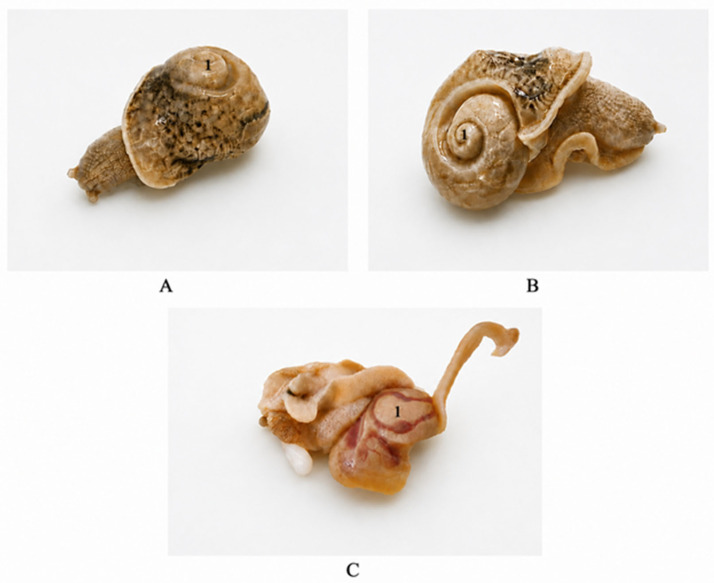
Anatomical localization of the hepatopancreas (1) in shell-removed *Helix pomatia*, showing its position within the apical region of the visceral cone in dorsal (**A**) and lateral (**B**) views and in dorsal view after removal of the mantle covering the visceral mass (**C**).

Approximately 3 kg of *H. pomatia* was obtained from snail-purchasing centres in each of two Polish voivodeships, Lower Silesia and Kuyavian–Pomeranian, during the regulated harvest season in Poland (20 April–31 May) (Figure 1). These regions were selected due to the availability of legally harvested *H. pomatia* and the presence of established snail-purchasing centres collecting specimens from broad geographical areas. In addition, the two voivodeships represent contrasting environmental and anthropogenic settings, enabling preliminary regional comparisons. Lower Silesia has a long history of intensive mining and metallurgical and energy-related industrial activity, which continues to shape its environmental characteristics, whereas Kuyavian–Pomeranian Voivodeship is characterised by a predominantly agricultural landscape with less intensive industrial development. The purchasing centres collected wild snails from multiple local collectors operating throughout each voivodeship during the harvesting season; consequently, the analysed material comprised snails originating from various locations within the respective regions. Only snails with a shell diameter > 30 mm and body weight > 15 g were included in the study. A total of 48 individuals were analysed, including 24 randomly selected specimens from each regional batch for laboratory processing. Specimens were sacrificed and dissected, and two anatomical fractions were separated: foot muscle and hepatopancreas. To isolate the hepatopancreas, the shell was removed, and the visceral mass was exposed. The hepatopancreas, located in the apical region of the visceral coil and surrounding the stomach and proximal intestine, was carefully separated from adjacent connective tissues and mantle using fine forceps and dissecting scissors, while avoiding damage to the digestive tract and gonad. The anatomical localisation of the hepatopancreas within the soft tissues of *H. pomatia* is shown in Figure 2. For each tissue type, pooled samples were prepared by combining material from eight individuals (*n* = 8), resulting in three composite samples (*n* = 3) per region and tissue. The pooling design was adopted to obtain composite samples representative of each regional batch while ensuring sufficient tissue mass for PCDD/F, PCB, and lipid analyses, particularly given the relatively small mass of the hepatopancreas, maintaining a feasible number of HRGC-HRMS determinations, and allowing assessment of variability among pooled samples within each regional batch. Each pooled sample was homogenised separately, and representative subsamples were taken for further analysis.

The analytical procedure used for the determination of PCDD/Fs and PCBs has been described previously [41]. The main methodological details relevant to the present study are summarised below.

### 2.2. Target Analytes, Standards, and Calibration

The analysis included 35 congeners: seven PCDDs (2,3,7,8-TCDD, 1,2,3,7,8-PeCDD, 1,2,3,4,7,8-HxCDD, 1,2,3,6,7,8-HxCDD, 1,2,3,7,8,9-HxCDD, 1,2,3,4,7,8-HpCDD, and OCDD), ten PCDFs (2,3,7,8-TCDF, 1,2,3,7,8-PeCDF, 2,3,4,7,8-PeCDF, 1,2,3,4,7,8-HxCDF, 1,2,3,6,7,8-HxCDF, 1,2,3,7,8,9-HxCDF, 2,3,4,6,7,8-HxCDF, 1,2,3,4,6,7,8-HpCDF, 1,2,3,4,7,8,9-HpCDF, and OCDF), twelve dl-PCBs (PCB 77, 81, 105, 114, 118, 123, 126, 156, 157, 167, 169, and 189), and six ndl-PCBs (PCB 28, 52, 101, 138, 153, and 180).

Five mixtures of internal standards for each compound group were prepared by dilution of commercially available standards. Each mixture contained a set of ^13^C_12_-labeled analogues of the analysed congeners. The concentrations of internal standards were 25 pg mL^−1^ for PCDD/PCDFs and 400 pg mL^−1^ for dl-PCBs and ndl-PCBs. ^13^C_12_-labelled 1,2,3,4-TCDD and PCB-111 were used as recovery control standards at concentrations of 25 and 400 pg mL^−1^, respectively. Calibration curves consisted of seven points for PCDD/Fs (0.05–5 pg µL^−1^), six points for dl-PCBs (0.02–40 pg µL^−1^), and five points for ndl-PCBs (1–500 pg µL^−1^). Good linearity was observed across all calibration ranges, with coefficients of determination (R^2^) exceeding 0.99 for all analytes.

### 2.3. Reagents and Chemicals

All solvents used were of analytical grade suitable for trace residue analysis. Hexane, dichloromethane, toluene, and *n*-nonane were obtained from LGC Standards (Wesel, Germany). Sodium sulphate and sulphuric acid (98%, ACS grade) were purchased from Merck (Darmstadt, Germany), while diatomaceous earth was supplied by Restek (Bellefonte, PA, USA). High-purity gases, including helium (99.9999%) and nitrogen (99.999%), were used for instrumental analysis. Native and isotopically labelled ^13^C_12_ PCDD/F and PCB standards were purchased from Cambridge Isotope Laboratories (Andover, MA, USA) and Wellington Laboratories (Guelph, ON, Canada).

### 2.4. Sample Extraction and Clean-Up

For the analysis of PCDD/PCDFs and PCBs, homogenised samples (an average of 20 g) were mixed with diatomaceous earth, and isotopically labelled internal standards were added prior to extraction. Sample extraction was performed using an ASE 350 accelerated solvent extractor (Dionex, Sunnyvale, CA, USA) with a hexane/dichloromethane (50:50, *v*/*v*) solvent mixture at 100 °C and 100 bar. The fat content was determined gravimetrically from an aliquot of the same extract used for PCDD/F and PCB analysis.

The obtained extracts were purified using an automated multi-column clean-up system (Power-Prep), involving chromatographic separation on modified silica, Florisil, and carbon-based sorbents to remove co-extracted interfering compounds. Purified fractions corresponding to PCDD/PCDFs and PCB groups were concentrated and prepared for instrumental analysis.

### 2.5. HRGC-HRMS Analysis

Analyses were performed using an Ultra Trace gas chromatograph (Thermo Scientific, Bremen, Germany) equipped with a CTC Analytics automatic sample injector and coupled to a MAT95XP high-resolution mass spectrometer (Thermo Scientific, Bremen, Germany). The mass spectrometer, featuring double focusing (magnetic and electrostatic) and inverted Nier–Johnson geometry, was operated in electron ionisation (EI) mode at a resolving power exceeding 10,000. Chromatographic separation was achieved using a capillary column suitable for dioxin analysis (e.g., DB-5MS, 60 m × 0.25 mm × 0.1 μm). The PCDD/PCDFs fraction and the PCBs fraction were analysed in two separate chromatographic runs. Quantification of PCDD/Fs and dl-PCBs was based on isotope dilution, using the ratio of native to corresponding ^13^C-labelled congeners monitored in selected ion mode.

### 2.6. Quality Assurance and Quality Control

A fully validated and accredited research method was used. Quality assurance and quality control (QA/QC) procedures included the use of isotopically labelled internal standards to determine recoveries (the recoveries ranged between 60% and 120%), analysis of procedural blanks (values in the blank sample below the method’s LOQ), and verification using certified reference materials (BCR-607, Geel, Belgium). LOD and LOQ values for individual congeners were estimated based on the analysis of ten spiked blank samples in accordance with the Guidance Document on the Estimation of LOD and LOQ for Measurements in the Field of Contaminants in Feed and Food [42].

Method performance was additionally confirmed through participation in an external proficiency testing scheme. In the year in which the snail samples were analysed, a beef liver matrix was used, and satisfactory z-scores were obtained (−0.4 for total PCDD/F + dl-PCBs, −0.4 for total dl-PCBs, and +0.1 for total ndl-PCBs).

### 2.7. Calculation and Expression of Results

Concentrations of PCDD/Fs and dl-PCBs were expressed as pg/g fresh weight (fw), whereas ndl-PCBs were expressed as ng/g fw. TEQs for PCDD/Fs and PCDD/Fs + dl-PCBs were calculated using 2005 WHO-TEFs, as applied in Commission Regulation (EU) 2023/915 [40]. In addition, TEQs were also calculated using 2022 WHO-TEFs as proposed by DeVito et al. [43]. Lower-bound (LB), middle-bound (MB), and upper-bound (UB) concentrations were calculated by assigning non-quantified congener (<LOQ) values of zero, one-half of the LOQ, and the LOQ value, respectively.

### 2.8. Statistical Analysis

Statistical analysis was performed using lipid content data. The dataset was based on pooled samples, with three independent pooled samples per anatomical part and region (*n* = 3; each pool composed of eight individual organisms). The obtained data were tested for normality using the Shapiro–Wilk test and for homogeneity of variances using Levene’s test. Differences in lipid content between foot muscle and hepatopancreas within each region were evaluated using Student’s *t*-test. Data are presented as mean (min–max). Differences were considered statistically significant at *p* < 0.05. All statistical analyses were performed using Statistica 13.3 software (TIBCO Software Inc., Palo Alto, CA, USA).

## 3. Results and Discussion

The concentrations of PCDD/Fs, dl-PCBs, and ndl-PCBs determined in the foot muscle and hepatopancreas of free-living *H. pomatia* collected from two Polish voivodeships (Kuyavian–Pomeranian and Lower Silesia) are presented in Table 1. Overall, only trace concentrations of selected PCDFs and dl-PCBs were detected in the hepatopancreas. All analysed PCDD congeners were below the LOQ. Among PCDFs, only 2,3,7,8-TCDF and 1,2,3,7,8-PeCDF were quantified. In snails from Kuyavian–Pomeranian, the mean concentration of 2,3,7,8-TCDF in the hepatopancreas was 0.163 pg/g fw (range: 0.120–0.220 pg/g fw), whereas in Lower Silesia it reached 0.062 pg/g fw (range: 0.046–0.080 pg/g fw). Similarly, 1,2,3,7,8-PeCDF was detected at concentrations of 0.047 pg/g fw (range: 0.040–0.060 pg/g fw) and 0.016 pg/g fw (range: 0.013–0.030 pg/g fw), respectively. PCB 77 was the only quantified dl-PCB congener and occurred exclusively in hepatopancreas samples from Kuyavian–Pomeranian, with a mean concentration of 2.497 pg/g fw (range: 2.140–2.850 pg/g fw). All remaining dl-PCBs and all analysed ndl-PCBs were below the LOQ.

Despite the contrasting levels of anthropogenic pressure between the two regions, no clear differences in the concentrations of the analysed compounds were observed. Lower Silesia is among the most industrialised regions of Poland and one of the leading regions in terms of industrial waste generation [44], suggesting that higher contamination levels might have been expected. However, this finding may be partly explained by the low detection frequency and the limited number of quantified congeners, which reduced the ability to identify subtle regional differences. Moreover, contamination levels in terrestrial gastropods are likely influenced by local environmental conditions and nearby emission sources rather than broad regional characteristics alone.

The low concentrations of PCDD/Fs and PCBs observed in the present study should also be interpreted in relation to the lipid content of the analysed tissues (Table 2). The foot muscle contained very low lipid levels, amounting to 0.15% (range: 0.14–0.16%) in snails from Kuyavian–Pomeranian and 0.10% (range: 0.09–0.11%) in snails from Lower Silesia. In contrast, the hepatopancreas contained significantly higher lipid levels than the foot muscle in both analysed regions (*p* < 0.001), reaching 0.50% (range: 0.48–0.52%) and 0.30% (range: 0.29–0.32%), respectively. Since PCDD/Fs and PCBs are highly lipophilic compounds, their preferential detection in the hepatopancreas is likely associated with the higher lipid content of this tissue. The exclusive detection of selected PCDFs and PCB 77 in the hepatopancreas therefore indicates that this organ represents the primary site of accumulation of lipophilic contaminants in *H. pomatia*.

In the absence of specific regulatory limits for PCDD/Fs and PCBs in terrestrial edible snails, and because information on PCDD/F occurrence in terrestrial gastropods is virtually unavailable, the obtained results were compared primarily with published data for aquatic gastropods and supplemented by evidence from studies on other soil-associated invertebrates. Accordingly, numerous studies have confirmed the presence of PCDD/Fs, dl-PCBs, and ndl-PCBs in marine and freshwater gastropods, with concentrations generally reflecting local contamination levels [22,23,24,25,26,27,28,29,30,31,32,33,34]. Comparative data reported in the literature are summarised in Table 3. Overall, the concentrations observed in the present study were markedly lower than those reported for many aquatic gastropods, particularly those collected from highly contaminated environments. For example, freshwater snails collected from Vietnamese dioxin hot spots exhibited WHO-TEQ values of up to 53.6 pg/g ww [29], whereas freshwater gastropods from an e-waste dismantling area in China contained 174–1296 pg/g dw of PCDD/Fs, with combined PCDD/F and dl-PCB WHO-TEQ values ranging from 14.3 to 347 pg/g dw [30]. Elevated PCB concentrations have also been reported in apple snails from e-waste recycling regions (3.78–1812 ng/g dw) [27], in marine gastropods collected from industrialised coastal areas (253–1001 ng/g lw) [28], and in freshwater gastropods collected near e-waste recycling sites (2.65–214 ng/g dw), where PCB levels reflected soil contamination, with elevated concentrations observed at e-waste sites and a decreasing trend over time [32]. Such elevated concentrations in aquatic gastropods are likely associated with continuous exposure to dissolved and sediment-associated contaminants, which may facilitate the uptake and trophic transfer of highly lipophilic POPs in aquatic food webs.

In contrast, terrestrial gastropods are exposed primarily through contact with contaminated soil and ingestion of plant material, where the bioavailability of highly hydrophobic contaminants may be lower than in aquatic systems. Among the limited data available for terrestrial gastropods, the accumulation of PCBs has recently been demonstrated in *Cornu aspersum* exposed under different environmental conditions in Algeria [37]. In that study, PCB concentrations in soft tissues ranged from 4.1 to 2780.9 ng/g wet weight depending on the sampling site and degree of anthropogenic pressure, with the highest concentrations observed in industrial areas. PCB concentrations were also strongly correlated with contamination levels in surrounding soils, indicating that soil represented the major source of exposure [37].

Compared with the concentrations reported in the Algerian study, the levels detected in the present investigation were markedly lower. However, direct comparison between the two studies should be made with caution due to substantial differences in study design and exposure conditions. In the study by Al-Alam et al. [37], caged *Cornu aspersum* were intentionally exposed at sites representing different degrees of anthropogenic pressure, including industrial areas, whereas the present investigation involved free-living *H. pomatia* collected under natural environmental conditions in Poland. Moreover, the observed differences may also reflect species-specific accumulation patterns, as terrestrial gastropods may differ in their ecological and physiological capacity to accumulate highly lipophilic POPs.

Beyond differences in contamination levels, the congener profile may also provide insight into the environmental behaviour, bioavailability, and uptake of individual compounds. The relatively limited ability of molluscs to metabolise and eliminate organic contaminants can favour their retention in tissues [45,46]. Consequently, the observed congener profile may be influenced by differences in bioavailability, degree of chlorination, and uptake characteristics [47,48,49,50]. In the present study, only the lower-chlorinated congeners 2,3,7,8-TCDF and 1,2,3,7,8-PeCDF, together with PCB 77, were detected, whereas most highly chlorinated congeners remained below the LOQ.

Similar relationships between chlorination degree and bioavailability have been reported for other soil-associated invertebrates. Shang et al. [51] demonstrated that the bioaccumulation of PCDD/Fs in earthworms decreased with increasing chlorination, indicating lower uptake of highly chlorinated congeners. Likewise, Manier et al. [45] reported lower accumulation of highly chlorinated PCDD/Fs following transfer from contaminated soils to earthworms (*Eisenia fetida*) and land snails (*Cantareus aspersus*). Henriksson et al. [52] showed that although field-collected earthworms contained high overall PCDD/F concentrations (290–520,000 pg/g fresh weight), bioaccumulation factors were generally lower for hepta- and octa-chlorinated homologues than for lower-chlorinated congeners, indicating greater bioavailability of tetra- and penta-chlorinated compounds. Taken together, these observations support the interpretation that the detection of only 2,3,7,8-TCDF, 1,2,3,7,8-PeCDF, and PCB 77 in the present study may reflect the higher bioavailability and uptake efficiency of lower-chlorinated compounds from soil.

To provide an integrated toxicological assessment of the detected congeners, the corresponding WHO-TEQ values for PCDD/Fs and combined PCDD/Fs + dl-PCBs are presented in Table 4. TEQ concentrations were derived using the 2005 WHO-TEFs currently applied in Commission Regulation (EU) 2023/915, while recalculated values based on the updated 2022 WHO-TEFs proposed by DeVito et al. [43] were additionally included for comparison. Because several congeners occurred at concentrations below the LOQ, lower-bound (LB), middle-bound (MB), and upper-bound (UB) approaches were applied to estimate the uncertainty associated with left-censored data.

The very low concentrations and limited number of detected congeners were reflected in correspondingly low TEQ values in all analysed samples. In the foot muscle, the middle-bound PCDD/F-TEQ amounted to 0.039 pg TEQ/g fw in both analysed regions, whereas the middle-bound combined PCDD/F + dl-PCB-TEQ reached 0.062 pg TEQ/g fw. Slightly higher values were observed in the hepatopancreas, consistent with the preferential accumulation of lipophilic contaminants in this tissue. Nevertheless, even under the upper-bound scenario representing the most conservative approach for handling congeners detected below the LOQ, TEQ values remained very low and did not exceed 0.138 pg TEQ/g fw in any analysed sample. Direct comparison with existing regulatory limits should be interpreted with caution, as these limits apply to other food categories, including fishery products and aquatic molluscs expressed on a fresh weight basis, as well as meat and certain animal by-products expressed on a fat basis. Nevertheless, the TEQ values determined in *H. pomatia* were consistently low in relation to the maximum levels established for other food products of animal origin. For example, even the highest upper-bound TEQ values observed in the present study were approximately 47-fold lower than the maximum level established for the sum of PCDD/Fs and dl-PCBs in fishery products and aquatic molluscs. Recalculation using the proposed 2022 WHO-TEFs did not substantially affect the interpretation of the results, as only minor differences between the two TEF systems were observed.

Finally, from a food safety perspective, the low TEQ values observed in the analysed *H. pomatia* samples suggest a limited toxicological relevance of PCDD/Fs and PCBs under the investigated conditions. Furthermore, all analysed compounds in the edible foot muscle were below the LOQ, whereas measurable concentrations were detected only in the hepatopancreas, a tissue that is normally removed prior to consumption in accordance with Regulation (EC) No. 853/2004 [39]. Consequently, consumer exposure associated with the consumption of properly processed *H. pomatia* is likely to be low. Nevertheless, a comprehensive dietary exposure assessment would require more extensive occurrence data together with reliable information on consumption patterns. Although terrestrial snails are regarded as a culinary delicacy and are consumed in several European countries, including Poland, reliable data on the consumption frequency and portion sizes of *H. pomatia* remain scarce, thereby limiting the development of realistic dietary exposure scenarios for this species.

Several limitations of the present study should be acknowledged. First, the study was limited to samples collected from two Polish voivodeships and therefore does not provide a comprehensive assessment of PCDD/F and PCB contamination in terrestrial snails across Poland. Second, most analysed congeners were detected at concentrations below the LOQ, and only a limited number of compounds were quantified. As a result, the ability to identify spatial patterns of contamination and potential accumulation trends was restricted. Third, the absence of environmental samples, such as soil or vegetation collected from the sampling areas, precluded direct assessment of relationships between contaminant levels in snails and environmental contamination. Therefore, further studies involving a larger number of sampling locations, broader geographical coverage, and complementary environmental matrices are warranted to better characterise spatial variation in contamination levels and the occurrence of PCDD/Fs and PCBs in free-living *H. pomatia*.

## 4. Conclusions

The present study found very low concentrations of PCDD/Fs and PCBs in free-living *H. pomatia* collected from two regions of Poland. Measurable concentrations of selected PCDFs and dl-PCBs were detected exclusively in the hepatopancreas, whereas all analysed compounds in the foot muscle remained below the limit of quantification. The exclusive detection of dioxin-like compounds in the hepatopancreas is consistent with the higher lipid content of this tissue and suggests that the hepatopancreas may represent an important site of accumulation of lipophilic contaminants in terrestrial snails. These findings are also consistent with the regulatory recognition of the hepatopancreas as a tissue of potential relevance for food safety, as reflected in Regulation (EC) No. 853/2004 [39].

Overall, the obtained TEQ values indicate a low toxicological burden in the analysed *H. pomatia* samples under the conditions investigated in this study.

To the best of our knowledge, the present study represents one of the first assessments of PCDD/Fs and PCBs in free-living *H. pomatia* and provides novel data on the occurrence of dioxin-like contaminants in this edible terrestrial snail species. Given the limited information currently available for terrestrial gastropods, the findings should be regarded as exploratory and interpreted with appropriate caution. Further investigations are needed to better characterise the environmental occurrence and accumulation of PCDD/Fs and PCBs in *H. pomatia*, as well as to evaluate the potential relevance of this species for environmental monitoring and food safety assessments.

## Figures and Tables

**Table 1:** Concentrations of individual PCDD/F (pg/g fw), dl-PCB (pg/g fw), and ndl-PCB (ng/g fw) congeners in the foot muscle and hepatopancreas of free-living *Helix pomatia* from two regions of Poland.

Congener	Limit of Quantification (LOQ)	Kuyavian–Pomeranian		Lower Silesia	
		Foot Muscle	Hepatopancreas	Foot Muscle	Hepatopancreas
PCDDs					
2,3,7,8-TCDD	0.01	˂LOQ	˂LOQ	˂LOQ	˂LOQ
1,2,3,7,8-PeCDD	0.01	˂LOQ	˂LOQ	˂LOQ	˂LOQ
1,2,3,4,7,8-HxCDD	0.02	˂LOQ	˂LOQ	˂LOQ	˂LOQ
1,2,3,6,7,8-HxCDD	0.07	˂LOQ	˂LOQ	˂LOQ	˂LOQ
1,2,3,7,8,9-HxCDD	0.02	˂LOQ	˂LOQ	˂LOQ	˂LOQ
1,2,3,4,7,8-HpCDD	0.35	˂LOQ	˂LOQ	˂LOQ	˂LOQ
OCDD	1.37	˂LOQ	˂LOQ	˂LOQ	˂LOQ
PCDFs					
2,3,7,8-TCDF	0.03	˂LOQ	0.163 (0.120–0.220)	˂LOQ	0.062 (0.046–0.080)
1,2,3,7,8-PeCDF	0.01	˂LOQ	0.047 (0.040–0.060)	˂LOQ	0.016 (0.013–0.030)
2,3,4,7,8-PeCDF	0.07	˂LOQ	˂LOQ	˂LOQ	˂LOQ
1,2,3,4,7,8-HxCDF	0.09	˂LOQ	˂LOQ	˂LOQ	˂LOQ
1,2,3,6,7,8-HxCDF	0.04	˂LOQ	˂LOQ	˂LOQ	˂LOQ
1,2,3,7,8,9-HxCDF	0.02	˂LOQ	˂LOQ	˂LOQ	˂LOQ
2,3,4,6,7,8-HxCDF	0.03	˂LOQ	˂LOQ	˂LOQ	˂LOQ
1,2,3,4,6,7,8-HpCDF	0.12	˂LOQ	˂LOQ	˂LOQ	˂LOQ
1,2,3,4,7,8,9-HpCDF	0.02	˂LOQ	˂LOQ	˂LOQ	˂LOQ
OCDF	0.13	˂LOQ	˂LOQ	˂LOQ	˂LOQ
dl-PCBs					
PCB 77	1.16	˂LOQ	2.497 (2.140–2.850)	˂LOQ	˂LOQ
PCB 81	0.09	˂LOQ	˂LOQ ^a^	˂LOQ	˂LOQ
PCB 126	0.31	˂LOQ	˂LOQ ^b^	˂LOQ	˂LOQ
PCB 169	0.38	˂LOQ	˂LOQ	˂LOQ	˂LOQ
PCB 105	15.83	˂LOQ	˂LOQ	˂LOQ	˂LOQ
PCB 114	2.83	˂LOQ	˂LOQ	˂LOQ	˂LOQ
PCB 118	16.85	˂LOQ	˂LOQ	˂LOQ	˂LOQ
PCB 123	0.92	˂LOQ	˂LOQ	˂LOQ	˂LOQ
PCB 156	16.52	˂LOQ	˂LOQ	˂LOQ	˂LOQ
PCB 157	5.58	˂LOQ	˂LOQ	˂LOQ	˂LOQ
PCB 167	10.06	˂LOQ	˂LOQ	˂LOQ	˂LOQ
PCB 189	2.77	˂LOQ	˂LOQ	˂LOQ	˂LOQ
ndl-PCB					
PCB 28	0.21	˂LOQ	˂LOQ	˂LOQ	˂LOQ
PCB 52	0.104	˂LOQ	˂LOQ	˂LOQ	˂LOQ
PCB 101	0.07	˂LOQ	˂LOQ	˂LOQ	˂LOQ
PCB 138	0.28	˂LOQ	˂LOQ	˂LOQ	˂LOQ
PCB 153	0.45	˂LOQ	˂LOQ	˂LOQ	˂LOQ
PCB 180	0.12	˂LOQ	˂LOQ	˂LOQ	˂LOQ

Results for each congener, anatomical part, and region are based on three pooled samples, each composed of eight individuals. Values are reported as <LOQ when all pooled samples were below the limit of quantification. When quantifiable values were obtained, results are presented as mean (min–max). For mixed datasets (quantified and <LOQ values), the middle-bound approach was applied, with values below the LOQ replaced by ½ LOQ. Lowercase letter (a) indicates that within the corresponding <LOQ result, one pooled sample showed a measured concentration above the LOQ (0.13 pg/g fw); lowercase letter (b) indicates the same for 0.34 pg/g fw.

**Table 2:** Lipid content (%) in foot muscle and hepatopancreas of free-living *Helix pomatia* from two regions of Poland.

Tissue	Voivodeship of Origin	Lipid Content	*p*-Value
Foot muscle	Kuyavian–Pomeranian	0.15 (0.14–0.16)	0.00001
Hepatopancreas	Kuyavian–Pomeranian	0.50 (0.48–0.52)	
Foot muscle	Lower Silesia	0.10 (0.09–0.11)	0.00004
Hepatopancreas	Lower Silesia	0.30 (0.29–0.32)	

Results are based on three pooled samples, each composed of eight individuals per anatomical part and region, and are expressed as mean (min–max). Differences between foot muscle and hepatopancreas within each region were assessed using Student’s *t*-test.

**Table 3:** Summary of published studies on PCDD/Fs and PCBs in gastropods.

Species	Country (Region/Area)	Habitat	Matrix	Compounds	Σ Concentration	WHO-TEQ	Key Findings	Reference
*Amphibola crenata*	New Zealand (North Island)	estuarine	soft tissue	PCBs	302/2256 ng/g lw; 17/124 ng/g dw	-	Mud snails accumulated PCB residues in a contaminated estuary	[22]
*Lymnea* sp.	United States (Alaska)	freshwater	soft tissue	PCBs	5.04 ng/g ww	-	Freshwater snails accumulated PCB congeners despite very low sediment concentrations	[23]
*Augur territella*, *Pila ampullacea*	India (Porto Novo and Cuddalore)	marine freshwater	edible part	PCBs	4.8/12 ng/g ww	-	Gastropods from South India were shown to contain detectable PCB residues	[24]
*Rapana venosa*, *Neptunea arthritica cumingii*, *Neverita didyma*	China (Bohai Sea coastline)	marine	soft tissue	PCBs	68–584 ng/g lw; 0.55–4.8 ng/g ww; 3–16 ng/g dw	-	PCB concentrations reflected local contamination sources, whereas PCDD/F levels were more uniform across sites	[25]
				dl-PCBs	2.721–18.663 ng/g lw	0.53–16 pg/g lw		
				PCDD/Fs	n.d.	0.05–44 pg/g lw; 0.0004–0.6 pg/g ww; 0.002–2.1 pg/g dw		
*Haliotis discus hannai*, *Haliotis gigantea*, *Batillus cornutus*	Korea (coastal region)	marine	soft tissue	PCDDs	n.d.	0.015–0.054 pg/g ww	Gastropods contributed minimally to dietary intake of PCDD/Fs and dl-PCBs	[26]
				PCDFs	n.d.	0.016–0.097 pg/g ww		
				dl-PCBs	n.d.	0.008–0.087 pg/g ww		
Apple snail (Latin name unspecified)	China (southeastern region)	freshwater	soft tissue	PCBs	3.78–1812 ng/g dw	-	Apple snails accumulated elevated PCB concentrations near e-waste dismantling sites and were proposed as bioindicators of POP contamination	[27]
*Hexaplex trunculus*	Italy (Mediterranean area)	marine	soft tissue	PCBs	253–1001 ng/g lw; 15.2–56.1 ng/g ww	-	PCB levels in gastropods reflected anthropogenic pressure and were generally within EU limits, with dl-PCB (TEQ) exceedances at highly contaminated sites	[28]
				dl-PCBs	77–271 ng/g lw; 4.6–15.2 ng/g ww	2.14–3.53 pg/g ww		
species unspecified	Vietnam	freshwater	edible part	2,3,7,8-TCDD	1.4 pg/g ww	-	Freshwater snails were identified as high-risk food due to elevated PCDD/F TEQ levels	[29]
				PCDD/Fs	n.d.	53.6 pg/g ww		
*Bellarnya purificata*	China (eastern region)	freshwater	muscle tissue	PCBs	0.17–1.6 × 10^6^ pg/g dw	-	Gastropods showed high PCB accumulation and moderate PCDD/F levels, with TEQs exceeding EU limits for aquatic biota and no evidence of trophic magnification	[30]
				PCDD/Fs	174–1296 pg/g dw	n.d.		
				PCDD/Fs + PCBs	n.d.	14.3–347 pg/g dw		
*Ampullaria gigas spix*	China (eastern region)	freshwater	muscle tissue	PCBs	0.51–14.9 × 10^5^ pg/g dw	-		
				PCDD/Fs	18.6–64 pg/g dw	n.d.		
				PCDD/Fs + PCBs	n.d.	2.77–5.69 pg/g dw		
*Bellamya aeruginosa*	China (eastern region)	freshwater	soft tissue	PCBs	90–680 ng/g lw	-	Snails showed moderate PCB levels, with CB-153 as the dominant congener; bioaccumulation was observed, supporting their suitability as biomonitors	[31]
*Pomacea canaliculata*	China (southern region)	freshwater	soft tissue	PCBs	2.65–214 ng/g dw	n.d.	Freshwater gastropods reflected soil PCB contamination, with elevated levels at e-waste sites and a decreasing trend over time	[32]
*Tegula atra*, *Prisogaster niger*, *Nucella* sp., *Collisella* sp., *Fissurella* sp., *Nassarius dentifer*, *Xanthochorus giganteus*, *Nassarius gayi*, *Oliva peruviana*, *Crassilabrum crassilabrum*, *Chorus giganteus*	Chile (southcentral region)	marine	soft tissue	PCDD/Fs	n.d.	up to 4.38 pg/g dw	Marine gastropods showed low PCDD/F levels, with no evident spatial pattern	[33]
*Sinotaia quadrata*	Italy (central region)	freshwater	edible part	ndl-PCBs	3.07/7.32 ng/g ww	-	Freshwater gastropods showed low ndl-PCB concentrations and no apparent human health risk	[34]
*Cornu aspersum*	Lebanon	terrestrial	soft tissue	PCBs	4.1–2780.9 ng/g ww	-	Levels correlated with soil contamination; site-dependent, with higher values at industrial sites compared to suburban and rural areas	[37]

Values are reported as single concentrations when one dataset (e.g., pooled sample or mean) was available. When two values were reported (e.g., for two sampling locations or species), individual results are presented. For datasets with more than two values, ranges (min–max) are shown. Ranges represent either variability within a study (as reported by the authors) or ranges derived from reported mean values or subgroup-specific data (e.g., different species or sampling locations). Concentrations are reported on a dry weight (dw), wet weight (ww), or lipid weight (lw) basis, as presented in the original studies; n.d. indicates no data available.

**Table 4:** TEQ values (pg TEQ/g fw) for PCDD/Fs and PCDD/Fs + dl-PCBs in foot muscle and hepatopancreas of free-living *Helix pomatia* from two regions of Poland.

Tissue	Voivodeship of Origin	PCDD/F-TEQ			PCDD/F + dl-PCB-TEQ		
		LB	MB	UB	LB	MB	UB
Foot muscle	Kuyavian–Pomeranian	0.000 (0.000)	0.039 (0.046)	0.079 (0.092)	0.000 (0.000)	0.062 (0.056)	0.123 (0.112)
Foot muscle	Lower Silesia	0.000 (0.000)	0.039 (0.046)	0.079 (0.092)	0.000 (0.000)	0.062 (0.056)	0.123 (0.112)
Hepatopancreas	Kuyavian–Pomeranian	0.017 (0.012)	0.055 (0.057)	0.093 (0.102)	0.018 (0.012)	0.078 (0.067)	0.138 (0.122)
Hepatopancreas	Lower Silesia	0.007 (0.005)	0.044 (0.049)	0.082 (0.094)	0.007 (0.005)	0.067 (0.060)	0.127 (0.115)

TEQ values were calculated using 2005 WHO-TEFs, as applied in Commission Regulation (EU) 2023/915 [40]. TEQ values based on 2022 WHO-TEFs, as proposed by DeVito et al. [43], are shown in parentheses. Lower-bound (LB), middle-bound (MB), and upper-bound (UB) approaches were applied for left-censored data by assigning non-quantified congeners (<LOQ) values of 0, ½ LOQ, and LOQ, respectively.

## Data Availability

The original contributions presented in this study are included in the article. Further inquiries can be directed to the corresponding author.
